# Impact of Diabetes-related Self-management on Glycemic Control in Type II Diabetes Mellitus

**DOI:** 10.7759/cureus.7845

**Published:** 2020-04-27

**Authors:** Khalid A Sayeed, Azwa Qayyum, Fatima Jamshed, Usman Gill, Syed Muhammad Usama, Kanza Asghar, Amber Tahir

**Affiliations:** 1 Internal Medicine, Liaquat College of Medicine and Dentistry, Darul Sehat Hospital, Karachi, PAK; 2 Pharmacology, Amna Inayat Medical College, Sheikhupura, PAK; 3 Pediatrics, Jinnah Sindh Medical University, Karachi, PAK; 4 Emergency Medicine, Hayyat Memorial Teaching Hospital, Lahore, PAK; 5 Emergency Medicine, Akhtar Saeed Medical College, Lahore, PAK; 6 Internal Medicine, Dow University of Health Sciences, Karachi, PAK; 7 Surgery, Hayyat Memorial Teaching Hospital, Lahore, PAK

**Keywords:** self-management, diabetes, glycemic control, glycated hemoglobin, hba1c, diabetes self-management questionnaire

## Abstract

Introduction: Self-care activities are behaviors adopted in order to enhance one’s health. Self-care behaviors and activities are studied in their role to enhance glycemic control, reduce diabetes-related complications, and contribute to enhancing overall quality of life in people with diabetes. The aim of this observational study was to evaluate the impact of diabetes self-care activities and behaviors on glycemic control in people with diabetes.

Methods: This observational, cross-sectional study was conducted at the outpatient department of a secondary care hospital in Karachi, Pakistan from 1st September 2019 till 30th November 2019. Patients with known type II diabetes of age ≥45 years visiting the hospital for routine follow-up visit were included. Diabetes Self-Management Questionnaire (DSMQ) in Urdu version was used to assess their status of self-management. For data entry and statistical analysis SPSS for Windows version 21.0 was used.

Results: There were 174 (54.9%) males and 152 (47.9%) were of age 45-60 years. Glycemic control was good (HbA1c <7%) in 125 (39.4%) and poor (HbA1c ≥7%) in 192 (60.6%) patients. Patients with good glycemic control scored significantly better on DSMQ overall (5.53 ± 0.35 vs. 4.32 ± 0.61; p<0.0001), and on three sub-scales - dietary control (4.24 ± 1.04 vs. 3.63 ± 0.98; p<0.0001), physical activity (4.16 ± 0.56 vs. 3.47 ± 1.17; p<0.0001), and healthcare use (4.22 ± 0.78 vs. 3.98 ± 0.65; p=0.003).

Conclusions: The self-care activities that impact glycemic control in patients with diabetes include dietary control, physical activity, and healthcare use.

## Introduction

For chronic illnesses including diabetes mellitus (DM), healthcare providers continue to struggle with the forever evolving needs of their patients. Keeping the patients connected to a healthcare facility through regular follow ups is crucial in instilling a sense of self-reliance in them and also in preventing long-term complications [1]. However, the management of diseases with multiorgan involvement such as DM is not limited to healthcare settings only. The holistic approach of DM management spans across dietary regulations, physical activity, medication compliance, and self-management [2].

According to the figures from American Diabetes Association (ADA), only one half of people with diabetes (PWD) are maintaining their glycated hemoglobin (HbA1c) at the recommended levels of <7% [3]. There are some demographic factors which impact the levels of HbA1c and cannot be altered such as gender, age, social, and educational status. However, there are also some modifiable psychosocial factors such as self-care activities and self-efficacy which in turn comes from disease-related education [4]. Self-care activities are the behaviors adopted in order to enhance one’s health [4]. In management of DM, role of self-care has been pivotal. It is an extremely essential component of holistic approach towards management of diabetes and can be achieved with a combination of awareness, knowledge, and internal motivation towards practices [4-5].

According to American Association of Diabetes Educators, self-care activities in PWD are assessed by seven major parameters including healthy diet, physical activity, regular blood sugar monitoring, medication adherence, effective approach to problem-solving, strong coping skills, and risk-reduction behaviors [6]. Literature has shown these seven self-care behaviors and activities to enhance glycemic control, reduce diabetes-related complications, and contribute to enhancing overall quality of life [7-11]. Exercise, blood sugar monitoring, and foot care are also recommended in all PWD as essential self-care habits to improve clinical and quality of life outcomes [7]. Along with these positive outcomes, a systematic review from Pakistan indicated improvement in cardiovascular risk factors and blood pressure with DM self-management [12].

In light of the existing literature, an observational study was conducted to evaluate the impact of diabetes self-care activities and behaviors on glycemic control in PWD.

## Materials and methods

This observational, cross-sectional study was conducted at the outpatient department of a secondary care hospital in Karachi, Pakistan. The study duration was from 1st September 2019 till 30th November 2019. It was conducted after attaining approval from the ethical review board.

In this study, patients of both genders, of age 45 years and above, known cases of type II DM visiting the hospital for routine follow-up visit were included. Patients with any of the diabetes-related complications, patients being seen in the emergency or in-patient department, patients who did not have at least one available record of HbA1c from the last six months, and patients who could not read and/or understand written Urdu were excluded. All patients were included after attaining informed consent.

In order to record patient information, a semi-structured questionnaire was constructed. It included patient gender, age, marital status, education, occupation, duration and treatment for DM, and their HbA1c record from the last six months. In order to assess their status of self-management, Diabetes Self-Management Questionnaire (DSMQ) in Urdu version was used. DSMQ has been designed to evaluate the relationship between patients’ activities of self-care and their glycemic control [13]. The reliability and validity of Urdu version of DSMQ has been established [14]. DSMQ has 16 items of self-care and is divided into four subscales - Glucose Management subscale (item 1, 4, 6, 10, 12), Dietary Control subscale (item 2, 5, 9, 13), Physical Activity subscale (item 8, 11, 15), and Healthcare Use subscale (3, 7, 14). Item 16 represents overall self-care. Each item is rated on a four-item Likert scale where 0 stands for “does not apply to me” and 3 stands for “applies to me very much.” For scoring, nine negatively phrased items are reversed. All scores were transformed to a 0-10 scale (raw score/maximum score*10). Higher scores indicate more effective self-care [13].

For data entry and statistical analysis SPSS for Windows version 21.0 (SPSS Inc., Chicago, IL, USA) was utilized. Patient characteristics were presented as frequency and percentages and compared using Chi square test. For DSMQ overall and its subscales mean and standard deviation (SD) were calculated and for comparison independent sample t-test was applied. The p value ≤0.05 was taken as significant. 

## Results

There were 317 PWD in the study. Among them, 174 (54.9%) were males and 152 (47.9%) were of age 45-60 years. Most of the participants were married (n=195; 61.5%), educated till university level (n=156; 49.2%), and had office-based jobs (n=137; 43.2%). In our study sample, 125 (39.4%) had good glycemic control (HbA1c <7%) and 192 (60.6%) had poor glycemic control (HbA1c ≥7%). Most of these patients had type II DM for 5-10 years (n=135; 42.5%) and the most common form of treatment was oral hypoglycemic agents (n=114; 35.9%). The patients were categorized according to their glycemic control and their characteristics are shown in Table 1.

**Table 1:** Baseline characteristics of the study participants (n=317).

Baseline characteristics	Glycemic control		p value
	Good glycemic control; (n=125; 39.4%)	Poor glycemic control; (n=192; 60.6%)	
Gender			
Male	71 (56.8%)	103 (53.6%)	0.58
Female	54 (43.2%)	89 (46.3%)	
Age			
45-60 years	72 (57.6%)	80 (41.7%)	0.005
> 60 years	53 (42.4%)	112 (58.3%)	
Marital status			
Single	18 (14.4%)	29 (15.1%)	0.04
Married	87 (69.5%)	108 (56.3%)	
Divorced	08 (6.4%)	16 (8.3%)	
Widow	12 (9.6%)	39 (20.3%)	
Education			
No schooling	02 (1.6%)	06 (3.1%)	0.36
Primary to secondary level	56 (44.8%)	97 (50.5%)	
University level	67 (53.6%)	89 (46.4%)	
Occupation			
Unemployed	07 (5.6%)	36 (18.7%)	0.004
Office job	54 (43.2%)	83 (43.2%)	
Outdoor job	39 (31.2%)	41 (21.4%)	
Stay at home	25 (20.0%)	32 (16.7%)	
Duration of diabetes			
< 5 years	43 (34.4%)	63 (32.8%)	0.04
5-10 years	61 (48.8%)	74 (38.5%)	
> 10 years	21 (16.8%)	55 (28.6%)	
Diabetes treatment			
Insulin therapy	48 (38.4%)	63 (32.8%)	0.09
Oral hypoglycemic agents	36 (28.8%)	78 (40.6%)	
Insulin + oral hypoglycemic agents	41 (32.8%)	51 (26.5%)	

In Table 2, the comparison of DSMQ scores between the two study groups is given. Patients with good glycemic control scored significantly better on DSMQ overall (5.53 ± 0.35 vs. 4.32 ± 0.61; p<0.0001), and on three sub-scales - dietary control (4.24 ± 1.04 vs. 3.63 ± 0.98; p<0.0001), physical activity (4.16 ± 0.56 vs. 3.47 ± 1.17; p<0.0001), and healthcare use (4.22 ± 0.78 vs. 3.98 ± 0.65; p=0.003). The scores on glucose management subscale were not significantly different in patients with good and poor glycemic control groups (Table 2).

**Table 2:** DSMQ overall and subscale scores of patients with good and poor glycemic control (n=317). DSMQ, Diabetes Self-Management Questionnaire; SD, standard deviation

DSMQ (mean ± SD)	Glycemic control		p value
	Good glycemic control; (n=125; 39.4%)	Poor glycemic control; (n=192; 60.6%)	
DSMQ overall score	5.53 ± 0.35	4.32 ± 0.61	<0.0001
Glucose management subscale	4.01 ± 0.44	3.87 ± 0.76	0.06
Dietary control subscale	4.24 ± 1.04	3.63 ± 0.98	<0.0001
Physical activity subscale	4.16 ± 0.56	3.47 ± 1.17	<0.0001
Healthcare use subscale	4.22 ± 0.78	3.98 ± 0.65	0.003

## Discussion

In this study, patients with good glycemic control scored significantly better on DSMQ overall and on three sub-scales - dietary control, physical activity, and healthcare use. The scores on glucose management subscale were not significantly different in patients with good and poor glycemic control groups.

In our study, 61% PWD had poor glycemic control (HbA1c >7%). The proportion is higher as compared to other local literature. In a study by Athar et al., 45% were reported to have HbA1c >8% and in a study by Malik et al., 53% had HbA1c >7% [15-16]. However, in a study by Bukhsh et al., as many as 83% PWD had poor glycemic control (HbA1c >7%) [11]. As far as international figures are concerned studies from Saudi Arabia and Jordan, 56% PWD had uncontrolled HbA1c [9, 17]. In Kuwaiti PWD, 75% were reported to have uncontrolled HbA1c [18].

In the present study, both groups were further categorized into strata according to their various demographic characteristics. It was observed that patients who were older than sixty years were more likely to have a poorer glycemic control compared to those who were younger (p<0.05). This could be because older individuals have impaired cognitive skills and as they age, their executive functioning starts to decline which is necessary to accomplish glycemic control. This finding is in accordance with the previously published literature [9, 11, 17]. However, other factors associated with advanced age cannot be neglected.

We observed that about half of the participants with good glycemic control had a high education status with a bachelor’s degree at the least. This finding is in coherence with a recent exploratory study published by Turrin and Trujillo, which reported that patients who were not able to measure their carbohydrate intake or calculate their insulin dose had a poorer glycemic control with a mean A1C of 8.0 + 1.4 percent, compared to those with a better understanding of their condition (p=0.04) [19]. The majority of their patients who scored higher on the diabetes numeracy test (DNT-15) also had an evidently high education status compared to those who did not score high on their DNT-15. Therefore, this suggests that there may be an indirect link at play between the education status of the patients and their ability to self-manage and attain satisfactory glycemic control. Previously, in a local study, the education status of the patients was associated with their diabetes-related self-care activities (p=0.005), thus cultivating a better glycemic control [11]. 

In the present study, it was observed that marital status was significantly associated with poor glycemic control. This can be explained by the lack of social support among those who were divorced or widowed, subsequently resulting in the impairment of self-efficacy and self-management of DM. A study by Gunggu et al. reported that among patients with DM, family support was found to be a strong predictor for optimal level of diabetes-related self-management, as the social support received by the patients improves their self-perception resulting in effectual self-management and better disease control [20]. 

In our study, it was found that a combination of oral hypoglycemic drugs and insulin was more effective in achieving a good glycemic control among patients (p=0.09). This finding is further reinforced by a recent report published by the National Program for Prevention and Control of Diabetes (NPPCD-2018), which is indicative of a negative correlation between poor glycemic control and using a combination of insulin and oral hypoglycemic drugs (p=0.001) [21]. 

The present study reported that about one-fourth patients with poor glycemic control were unemployed. They also scored significantly low on their overall DSM questionnaire. We can speculate that the patients who were unemployed were unable to afford basic healthcare facilities including access to medication as indicated by their low mean score on the healthcare use subscale, henceforth, resulting in poor glycemic control. Rak et al., in their work reported that there was an indirect relationship between efficacy of self-management of diabetes and unemployment among his study population. Although he did not find any direct link between the two variables, it was interesting to observe that patients who were employed were more physically fit and their activities of daily living were less likely to be affected by their condition [22].

Diabetes self-management education and awareness has been shown to improve the patient outcome among patients with diabetes. According to a systematic review , it was reported that the A1C levels improved significantly among 60% of the patients who received diabetes self-management education [23]. It is recommended that patients with DM should be offered diabetes self-management education to boost-up their self-efficacy, self-perception, and to develop strong diabetes-related coping strategies in order to improve the glycemic control among the patients.

The current study has many strengthening points that makes it an invaluable contribution to the scientific community, nevertheless, it is not completely devoid of limitations. Language barriers were faced while recruiting the patients for the study. As the validated Urdu version of DSMQ was used, the patients who did not speak Urdu language were inevitably excluded from the study. Since, self-management techniques differ significantly by ethnicity, we cannot generalize the current study to all ethnicities of Pakistan. Further research should evaluate the diabetes-related self-management and ethnic disparities.

## Conclusions

The present study indicates that the diabetes-related self-management was directly linked with glycemic control among patients. Patients who had scored badly on DSMQ had poorer glycemic control compared with patients who scored higher on DSMQ. Age, marital status, employment status, duration of illness, and anti-glycemic therapy were significantly associated with the glycemic control among patients. It is essential to promote self-management education related to DM as a strategy to improve self-management and improve the overall patient outcome. 
